# MiRNA-146b-5p upregulates migration and invasion of different Papillary Thyroid Carcinoma cells

**DOI:** 10.1186/s12885-016-2146-z

**Published:** 2016-02-16

**Authors:** Cilene Rebouças Lima, Murilo Vieira Geraldo, Cesar Seigi Fuziwara, Edna Teruko Kimura, Marinilce Fagundes Santos

**Affiliations:** Department of Cell and Developmental Biology, Institute of Biomedical Sciences, University of São Paulo, Avenida Professor Lineu Prestes 1524, Prédio I, CEP 05508-000 São Paulo, SP Brazil

**Keywords:** MicroRNAs, Thyroid, Cancer, Invasion, PTC, Cell migration, miR-146b

## Abstract

**Background:**

Tumor invasiveness is directly related to the ability of tumor cells to migrate and invade surrounding tissues, usually degrading extracellular matrix. Despite significant progress in the knowledge about migration and invasion, there is much more to elucidate about their regulatory mechanisms, especially in cancer cells. MicroRNAs (miRs) were recently described as important regulators of migration. Differential expression of miRs in cancer is frequently associated with progression, invasion and metastasis. In papillary thyroid carcinoma (PTC), miR-146b-5p is highly expressed and positively correlated to the degree of malignancy.

**Methods:**

This study aimed to investigate the role of miR-146b-5p on the migratory and invasive behaviors of thyroid cells, using a non tumor rat thyroid follicular cell line (PCCl3) transfected with the miR-146b-5p genomic region, and two PTC cell lines (TPC-1 and BCPAP, bearing distinct oncogenic backgrounds), which express high levels of miR-146b-5p, after miR-146b inhibition by antagomiR and miR-146b overexpression by mimics-miR. Migration and invasion were studied by time-lapse and transwell assays (with and without Matrigel®). Gelatin degradation assays were also employed, as well as F-actin staining.

**Results:**

Migration and invasion of PCCl3 were increased 2-3x after miR-146b-5p overexpression (10X) and large lamellipodia were evident in those cells. After miR-146b-5p inhibition, TPC-1 and BCPAP migration and invasion were significantly reduced, with cells showing several simultaneous processes and low polarity. Gelatin degradation was inhibited in TPC-1 cells after inhibition of miR-146b-5p, but was unaffected in BCPAP cells, which did not degrade gelatin. The inhibition of miR-146b-5p in PCCl3 also inhibited migration and invasion, and additional (exogenous) overexpression of this miR in TPC-1 and BCPAP cells increased migration and invasion, without effects on cell morphology or gelatin degradation. The overexpression of SMAD4 in BCPAP cells, a validated target of miR-146b-5p and key protein in the TGF-β signaling pathway, inhibited migration similarly to the effects observed with the antagomiR 146b-5p.

**Conclusions:**

miR-146b-5p positively regulates migration and invasion of thyroid normal and tumor follicular cells (independently from their original mutation, either BRAF or RET/PTC), through a mechanism that involves the actin cytoskeleton but not an increased capacity of matrix degradation.

**Electronic supplementary material:**

The online version of this article (doi:10.1186/s12885-016-2146-z) contains supplementary material, which is available to authorized users.

## Background

Tumor invasiveness is directly related to the ability of tumor cells to migrate and invade surrounding tissues, spreading via blood and lymphatic circulation. In tumors, the more pronounced is the migratory phenotype, the higher is its metastatic potential [1]. A complex signal transduction network involving different pathways directly and indirectly controls tumorigenesis and invasion [2].

Highly invasive adherent tumor cells present a mesenchymal phenotype and are able to migrate faster, degrading extracellular matrix on their way. In general, in order to migrate, these cells polarize and form lamellipodia at the cell front, which are large membrane projections rich in branching actin filaments and lacking organelles. New adhesions to the extracellular matrix (ECM) are established, and some of them mature to become anchorage junctions to the actin cytoskeleton. Adhesion maturation is followed by the pulling of the cell body forward and retraction of the rear, partially due to the contraction of actin-myosin II bundles present inside the cell and in the cell cortex [3]. Sometimes filopodia, which are thin spike-like exploratory processes, precede or accompany lamellipodia formation. This migration cycle is regulated by Rho GTPases, central modulators of the cytoskeleton involved in many signaling pathways [4]. The classic regulatory cycle of Rho GTPases involve molecules that regulate GTP binding and hydrolysis, as well as the availability of GTPases to be activated, usually in cell membranes. In the last few years, other important regulatory mechanisms were described, including microRNAs (miRs) [5].

MicroRNAs are small, non-coding RNAs that regulate protein expression and have been implicated in both the promotion and suppression of metastasis [6]. The term ‘metastamir’ describes miRs that are involved in tumor metastasis in different ways, acting either as prometastatic or antimetastatic [7]. The role of miRs is post-transcriptional gene regulation via perfect or imperfect pairing with the 3’ untranslated region (UTR) of target messenger RNAs (mRNAs), leading to mRNA degradation or translation blockage. In tumors, the differential expression of miRs (up or down) is frequently associated with progression, invasion and metastasis. For this reason, miRs have been considered as potentially important tumor hallmarks, and their deregulation is the focus of studies that intend to find tools for early diagnosis, prognosis, monitoring and treatment [6, 7].

An example of tumor which invasive behavior is much less understood than its development is the Papillary Thyroid Carcinoma (PTC). Both in tumor progression and invasiveness, however, miRs are involved [8]. PTC is the most common thyroid type of cancer, representing about 80 % of all malignant tumors in this organ [9, 10]. It is usually a multifocal intra-thyroid tumor (65 % of cases), which can be encapsulated or infiltrative. Small localized PTCs show a 99 % survival rate at 20 years, being considered low risk cancers. Considering the scores usually applied to classify PTCs as low risk, such as age, grade, extent (invasiveness and distant metastasis), size, sex and nodal spread, about 80–85 % of PTCs have excellent prognosis. These scores, however, are not suited to predict tumor recurrence, which is common (up to 30 % of patients). PTC recurrence may occur up to 20 years after the initial diagnosis and is commonly associated with cervical lymph node metastasis [11, 12].

Very important genetic alterations involved in PTC development include RET/PTC rearrangements and BRAF^V600E^ mutation; RAS mutation is less common [13]. These mutations constitutively activate the same signaling pathway, and rarely overlap. The growth factor TGF-β is a negative regulator of thyroid follicular cell growth, and the evasion of TGF-β signaling by follicular thyroid cells results in increased proliferation, the acquisition of an invasive phenotype and tumor progression [14, 15].

Several large-scale studies have shown deregulation of miRs in thyroid tumor samples, when compared to normal thyroid tissues [6, 16–23]. The miR146b-5p has received great attention for being one of the most expressed and positively correlated with tumor aggressiveness and extra-thyroid invasiveness [24–26]. It has been investigated as a potential molecular marker, but its functional roles are still poorly understood [27, 28].

In this study, we aimed to investigate the influence of miR-146b-5p on the migratory and invasive behaviors of thyroid normal and tumor cells, in order to determine its contribution to thyroid carcinoma invasiveness. Experimental procedures were performed with non-tumor rat thyroid follicular cell line (PCCl3) and two PTC cell lines (TPC-1 and BCPAP) after overexpression and inhibition of miR-146b-5p by specific oligonucleotides, mimics-miR and antagomiR, respectively . Briefly, we found that the overexpression of miR-146b-5p in PCCl3 increased migration and invasion, whereas an opposite proportional effect was observed in PTC tumor cell lines after miR-146b-5p inhibition. The inhibition of miR-146b-5p in PCCl3 also inhibited migration and invasion, and exogenous overexpression of this miR in TPC-1 and BCPAP cells increased migration and invasion even more. The overexpression of SMAD4 in BCPAP cells, a validated target of miR-146b-5p and key protein in the TGF-β signaling pathway, inhibited migration similarly to the effects observed with the antagomiR 146b-5p. Curiously, migration was equally affected in the presence or absence of basement membrane (Matrigel®), and was not related to the cell’s capacity to degrade ECM. These results suggest that miR-146b-5p may be considered an important prometastatic metastamiR in PTC.

## Results

### Inhibition of miR-146b-5p decreases migration and invasion of the human papillary thyroid carcinoma (PTC) cell lines TPC-1 and BCPAP, whereas its overexpression increases both processes

**Fig. 1:**
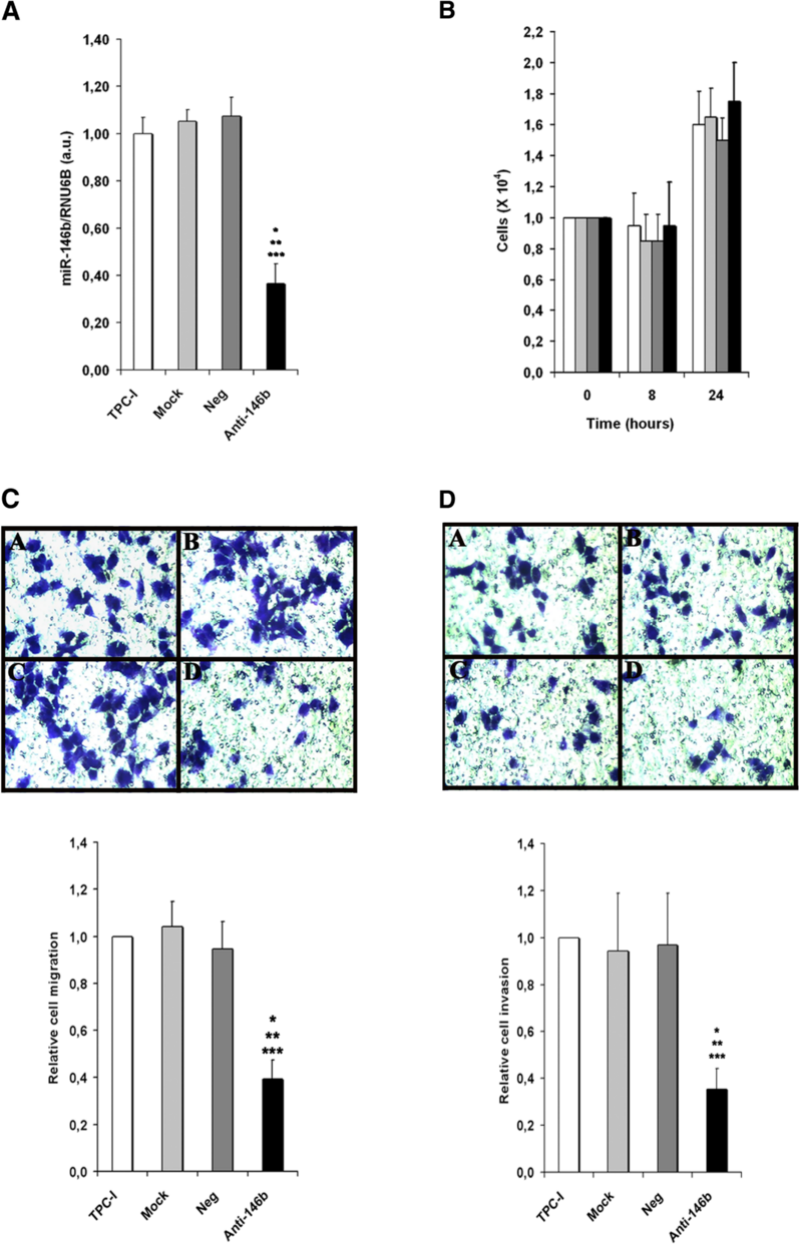
Inhibition of miR-146b-5p decreases migration and invasion of the PTC cell line TPC-1. Cells were transfected with an oligonucleotide antagomiR-146b-5p (Anti-146b) (30nM), as described in the Methods section. Three control groups were used: (1) cells cultured in regular culture medium (identified as TPC-1), (2) cells incubated with the transfection agent only (Mock) and (3) cells transfected with a negative miR-control (Neg). Forty-eight hours after transfection miR-146b-5p expression (**a**) and cell viability (**b**) were evaluated. Transwell migration (without basement membrane) and invasion (with basement membrane) assays were performed for 8 h. Representative images and quantitative data are shown for migration (**c**) and invasion assays (**d**). All experiments were performed three times. TPC-1: cell, Mock: cell + transfection agent, Neg: cell + anti-miR negative control, Anti-146b: cell + anti-miR-146b-5p. Statistically significant differences: * *P* < 0,01 (TPC-1 versus Anti-146b); ** *P* < 0,01 (Mock versus Anti-146b), *** *P*< 0,01 (Neg versus Anti-146b)

**Fig. 2:**
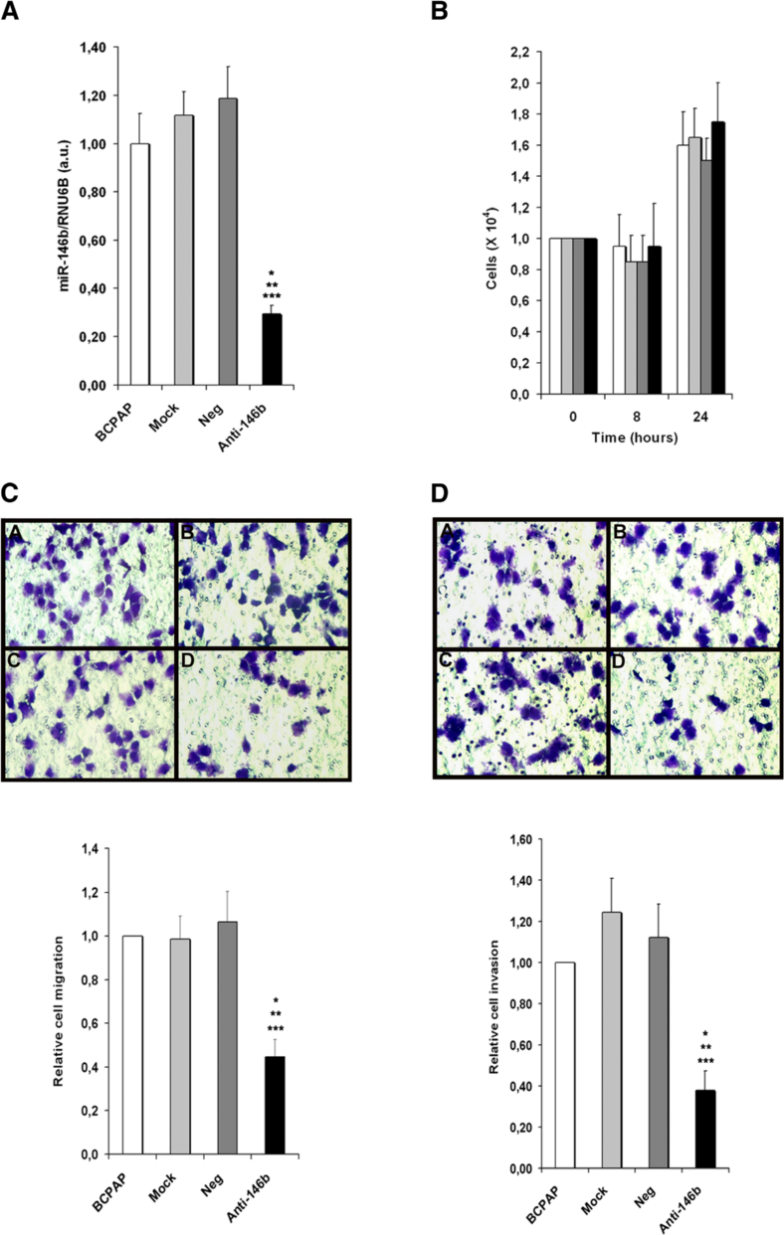
Inhibition of miR-146b-5p decreases migration and invasion of the PTC cell line BCPAP. Cells were transfected with an oligonucleotide antagomiR-146b-5p (Anti-146b) (30nM), as described in the Methods section. Three control groups were used: (1) cells cultured in regular culture medium (identified as BCPAP), (2) cells incubated with the transfection agent only (Mock) and (3) cells transfected with a negative miR-control (Neg). Forty-eight hours after transfection miR-146b-5p expression (**a**) and cell viability (**b**) were evaluated. Transwell migration (without basement membrane) and invasion (with basement membrane) assays were performed for 8 h. Representative images and quantitative data are shown for migration (**c**) and invasion assays (**d**). All experiments were performed three times. BCPAP: cell, Mock: cell + transfection agent, Neg: cell + anti-miR negative control, Anti-146b: cell + anti-miR-146b-5p. Statistically significant differences: * *P* < 0,01 (BCPAP versus Anti-146b); ** *P* < 0,01 (Mock versus Anti-146b), *** *P* < 0,01 (Neg versus Anti-146b)

MiR-146b-5p, which has been positively correlated to the degree of malignancy, is overexpressed in both human PTC cell lines, TPC-1 (spontaneously harboring the RET/PTC-1 mutation) [29] and BCPAP (BRAF^V^^600E^ oncogene point mutation) [30]. A specific miR-146b-5p oligonucleotide inhibitor (antagomiR) was used to reduce its expression in both cell lines (Figs. 1a and 2a) before the transwell migration and invasion assays. The results show that miR-146b-5p inhibition (~70 %) reduced by at least 50 % the migration and invasion capacity of TPC-1 (Fig. 1c and d) and BCPAP (Fig. 2c and d) cells. This result was not influenced by differences in cell number, as shown by cell viability assays (Figs. 1b and 2b).

The overexpression of miR-146b-5p in TPC-1 and BCPAP cells (by 11-12x), on the other hand, increased migration and invasion by 2–2.5x in both cell lines (Additional file 1: Figure S1).

### MiR-146b-5p inhibition reduced gelatin degradation by TPC-1, but not by BCPAP cell lines

**Fig. 3:**
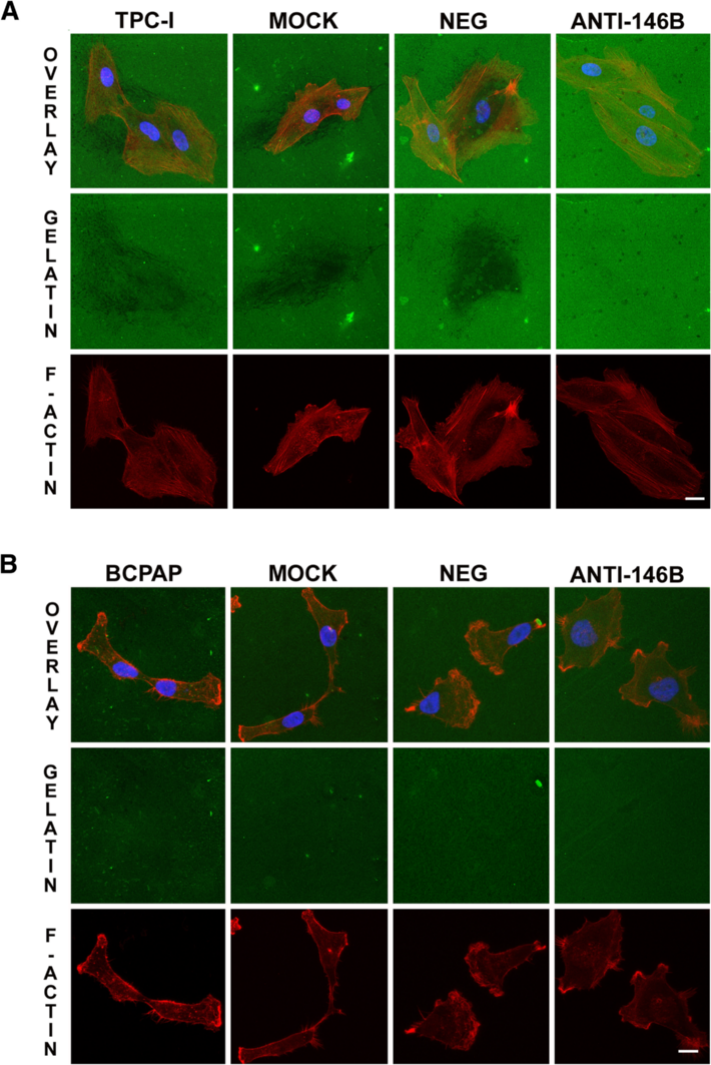
Inhibition of miR-146b-5p decreased gelatin degradation by TPC-1 cells. Forty hours after transfection, cells were seeded upon glass coverslips (18 mm) coated with fluorescent gelatin (*green*) and cultured for 8 h. After this period, cells were fixed, stained for F-actin (*red*) and nucleus (*blue*) and analyzed by confocal microscopy. Representative images are shown for TPC- 1 (**a**) and BCPAP (**b**) cells. The degradation activity of control and treated groups (miR-146b-5p inhibited) are identified as dark areas on gelatin-FITC background. TPC-1 / BCPAP: cell, Mock: cell + transfection agent, Neg: cell + anti-miR negative control, Anti-146b: cell + anti-miR-146b

In order to evaluate whether miR-146b-5p influences the extracellular matrix degradation during the invasion process, we used fluorescent gelatin degradation assays for both tumor cell lines, as shown in Fig. 3. Curiously, only the TPC-1 cell line degraded gelatin. After miR-146b-5p inhibition, the substrate degradation activity of this cell line was reduced to small focal points during the assay, very different from the control groups (Fig. 3a). In BCPAP cell line, no differences between controls and miR-146b-5p-inhibited groups were observed (Fig. 3b). The overexpression of miR-146b-5p in TPC-I cells led to a slight (not significant) increase in gelatin degradation, whereas BCPAP cells were still not able to degrade gelatin (Additional file 2: Figure S2).

### MiR-146b-5p inhibition reduced cell polarization and increased the number of cellular protrusions in TPC-1 and BCPAP cell lines

**Fig. 4:**
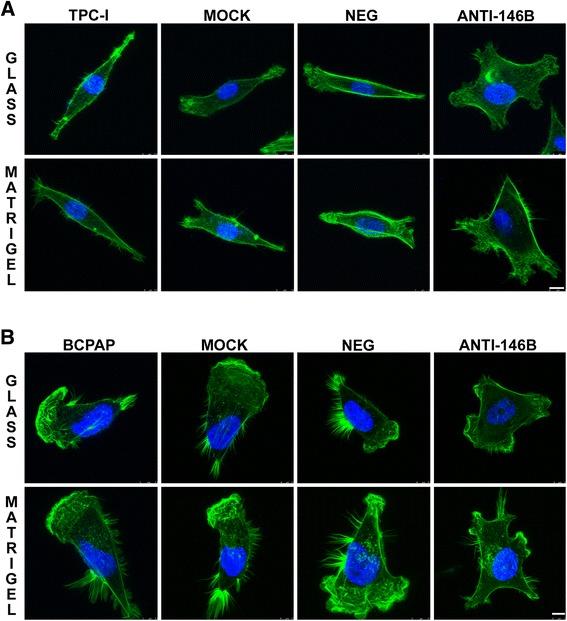
Inhibition of miR-146b-5p alter F-actin cytoskeleton distribution and cell morphology. Forty hours after transfection, cells were seeded upon glass coverslips (18 mm) without and with Matrigel® coating (10 μg/ml) and cultured for 8 h. After this period, cells were fixed, stained for F-actin (*green*) and nucleus (*blue*) and analyzed by confocal microscopy. Representative images of TPC-1 (**a**) and BCPAP cells (**b**) are shown. For both cell lines, control cells (TPC-1/BCPAP, Mock and Neg) are polarized and show one or two predominant lamellipodia, whereas cells with inhibition of miR-146b-5p (Anti-146B) show a higher number of smaller protrusions in different directions. TPC-1/BCPAP: cell, Mock: cell + transfection agent, Neg: cell + anti-miR negative control, Anti-146b: cell + anti-miR-146b. Bars: 10 μm

In order to investigate the effects of miR-146b-5p inhibition on cell morphology and actin cytoskeletal reorganization, we stained the F-actin filaments in both tumor cell lines using fluorescent phalloidin, as shown in Fig. 4. We observed that control cells (which express high levels of miR-146b-5p) were elongated, suggesting polarization, featuring one or two predominant lamellipodia. Additionally, BCPAP cells showed very large lamellipodia and many filopodia. In contrast, both TPC-1 and BCPAP cells with miR-146b-5p inhibited lost polarity, showing several protrusions in different directions, apparently smaller lamellipodia (Fig. 4). By time-lapse we observed that many of these cells did not translocate effectively due to this poorly polarized phenotype (data not shown).

Interestingly, the overexpression of miR-146b-5p in TPC-1 and BCPAP cells did not affect cell morphology or the F-actin distribution in both cell lines (Additional file 3: Figure S3). Probably these highly migratory cells were already optimized for effective migration, showing distinct polarization and one or two large lamellipodia.

### Overexpression of miR-146b-5p in normal rat thyroid cell line (PCCl3) increases migration and invasion, while its inhibition inhibits both processes

**Fig. 5:**
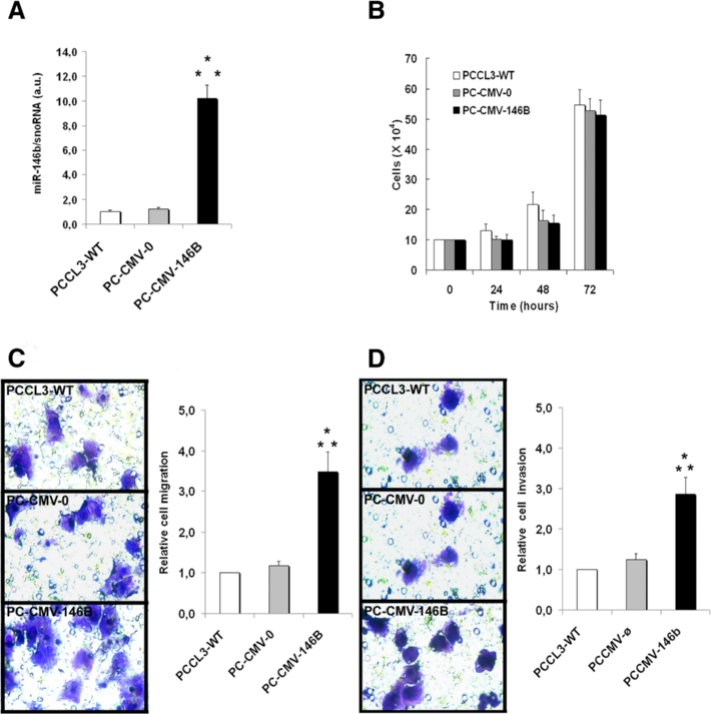
Overexpression of miR-146b-5p promotes migration and invasion of non tumor rat thyroid follicular cell line (PCCl3). Cells overexpressing miR-146-5p (PC-CMV-146b) and control groups (PC-CMV-ø and PCCl3-WT) were cultured and submitted to transwell migration and invasion assays for 24 h. After this period, miR-146b-5p expression (**a**) and cell viability (**b**) were evaluated. Representative images and quantitative data for migration (**c**) and invasion (**d**) assays are shown. PCCl3-WT: normal thyroid follicular cell line, PC-CMV-ø: cell transfected with an empty vector, PC-CMV-146b: cell transfected with the miR-146-5p genomic region. Statistically significant differences: * *P* < 0,01 (PCCl3-WT versus PC-CMV-146b); ** *P* < 0,01 (PC-CMV-ø and PC-CMV-146b)

**Fig. 6:**
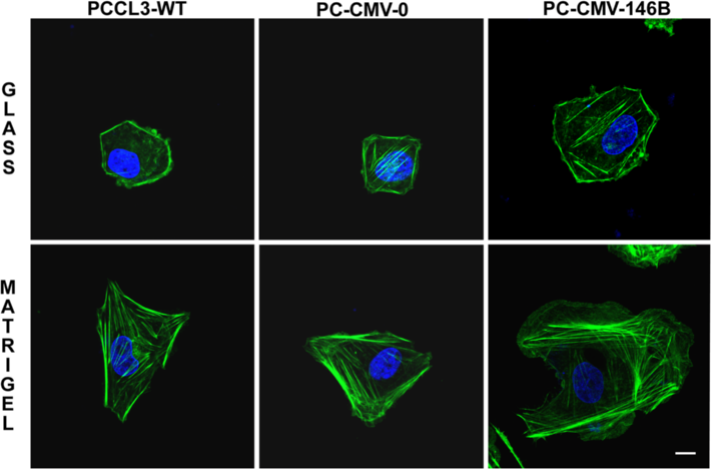
Overexpression of miR-146b-5p enhances cell spreading and formation of large lamellipodia in non tumor rat thyroid follicular cell line (PCCl3). Cells were seeded upon glass coverslips (18 mm) without and with Matrigel® coating (10 μg/ml) and cultured for 24 h. After this period, cells were fixed, stained for F-actin (*green*) and nucleus (*blue*) and analyzed by confocal microscopy. PCCl3-WT: non tumor rat thyroid follicular cell line, PC-CMV-ø: cell transfected with an empty vector, PC-CMV-146b: cell transfected with the miR-146-5p genomic region. Bars: 10 μm

Using a non tumor thyroid cell line (PCCl3) we aimed to evaluate if the overexpression of miR-146b-5p influences migration and invasion per se. We transfected the cells with a pcDNA3.1 plasmid containing the genomic region of miR-146b-5p (PC-CMV-146b) [31]. As controls, non transfected cells or cells transfected with the empty vector (PC-CMW-ø) were used. MiR-146b-5p expression was measured by qPCR. The results show that cells overexpressing miR-146b-5p (~10 times, Fig. 5a) migrated and invaded about 3 times more than control cells (Fig. 5c and d). No differences were observed in the growth curve up to 72 h (Fig. 5b). F-actin staining showed increased cell spreading, especially on Matrigel®, as well as the presence of large lamellipodia and more actin-myosin bundles (stress fibers) when compared to control cells (Fig. 6). On the other hand, the inhibition of miR-146b-5p by ~50–60 % using a specific antagomiR also inhibited migration and invasion by approximately 50–60 % (Additional file 4: Figure S4).

### Overexpression of SMAD4 in the BCPAP tumor cell line decreases migration and invasion

**Fig. 7:**
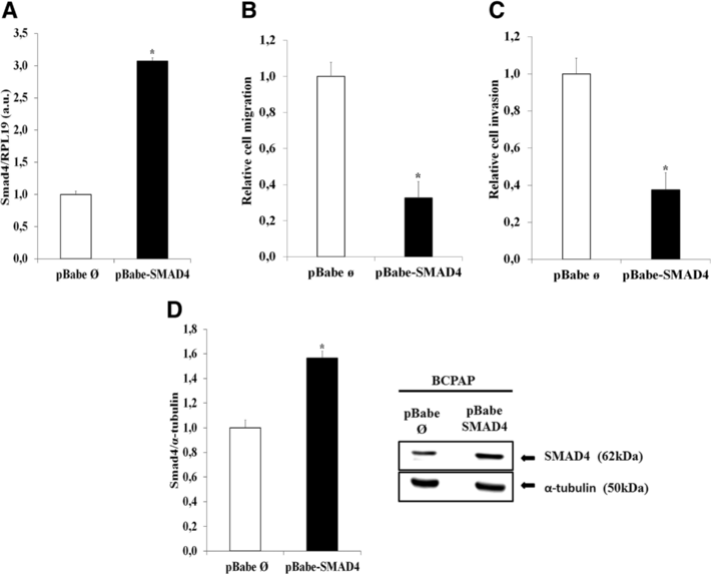
Overexpression of SMAD4 decreases migration and invasion of BCPAP cells. Cells were transfected with pBabe-puro-Smad4-Flag and pBABE-puro plasmids (control) as described in the Methods section. **a** The SMAD4 gene expression was analyzed by qPCR. RPL19 gene expression was used for normalization. **b**, **c** Transwell migration (without basement membrane) and invasion (with basement membrane) assays were performed during 8 h. pBabe ø: BCPAP transfected with pBABE-puro plasmids; pBabe SMAD4: BCPAP transfected with pBabe-puro-Smad4-Flag. **d** For SMAD4 protein expression, 50 μg of total protein per sample were used. α -Tubulin expression was used for normalization. Statistically significant differences: * *P*< 0,01 (pBabe SMAD4 versus pBabe ø)

BCPAP cells overexpressing SMAD4 were created by using BCPAP-pBabe SMAD4 plasmid and its respective control. The effectiveness of transfection was checked by RT-PCR (3x increased in the mRNA) and by western blotting (60 % increase in SMAD4 protein). BCPAP cells express high levels of miR-146-5p, which reduces SMAD4 basal expression [31]. Cells transfected with SMAD4 showed a 60–70 % decrease in migration and invasion, an effect very similar to the effect obtained after treatment with the antagomiR (Fig. 7).

## Discussion

A differential overexpression of miR-146b has been observed in different types of cancer, not always correlated with tumor progression or invasion [32–34]. Actually, regarding invasiveness, the miR-146b has been considered inhibitory of migration and invasion in several types of tumors, i.e. gliomas, lung cancer, pancreatic cancer, and osteosarcoma [32–37]. On the other hand, the high expression of miR-146b-5p in PTC has been positively correlated with the malignancy and aggressiveness in clinicopathological correlative studies [8, 38, 39]. It has also been associated with a higher degree of extra-thyroid invasiveness [10, 24–26]. Recently, using in vitro wound healing assays, Wojtas et al. [40] showed that the overexpression of miR-146b improved migration of HTori-3 and FTC-133 cell lines. In gliomas, reduced expression of miR-146b contributes to increase MMP16 and promote metastasis [37]. In thyroid cancer cells, the targeting of ZNFR3 by miR-146b stimulates Epithelial-to-mesenchymal transition (EMT) [39]. Nevertheless, the functional roles of miR-146b appear to affect different cellular processes important for tumor development and are still poorly understood.

In this study, we demonstrate that miR-146b-5p stimulates the migratory and invasive behavior of PTC cell lines. Overexpression of miR-146b-5p in a normal rat thyroid cell line (PCCl3) led to increased spreading on the substrate, formation of large lamellipodia, increased migration (without exogenous basement membrane) and invasion (in the presence of basement membrane). The opposite effect was observed after the inhibition of this miR in this cell line and also in human thyroid tumor cell lines which, although bearing different mutations, overexpressed miR-146b. Both TPC-1 and BCPAP cells showed decreased migration and invasion, lacking directionality due to the formation of smaller protrusions in different directions. Exogenous overexpression of miR-146b-5p in both tumor cell lines increased migration and invasion even more, without effects on cell morphology or in the F-actin arrangement.

TPC-1 and BCPAP cells are highly migratory [41, 42]. In both tumor cell lines, our results showed a similar degree of inhibition for migration and invasion, suggesting that the effects of miR-146b-5p are mostly associated with the cytoskeleton, and not necessarily related to increased degradation of ECM. This hypothesis was further confirmed by gelatin degradation assays, which showed that only TPC-1 significantly degraded gelatin under regular conditions. The inhibition of miR-146b-5p significantly reduced this function in those cells, although without any additional disadvantage to the cells to migrate. Besides, it was interesting to observe that the inhibition of migration was similar in both cell lines, considering that their proteolytic capacities may be quite different. The overexpression of miR-146b-5p slightly increased gelatin degradation by TPC-1 cells, having no effect on BCPAP cells gelatinolytic activity.

In several carcinomas, different signaling pathways such as TGF-β, Wnt-β-catenin and Notch are involved in Epithelial-Mesenchymal Transition (EMT), a crucial step for invasion of surrounding tissues [43]. SMAD4, a crucial protein in the canonical TGF-β signaling pathway, was validated as a target of miR-146b-5p in BCPAP, TPC-1 and PCCl3 cells [31]. The authors showed that miR-146b-5p targets the 3'UTR of SMAD4. Interestingly, in PCCl3 normal thyroid cells the activation of the oncogenes RET/PTC3 and BRAF upregulated miR-146b-5p expression [31].

In thyroid gland homeostasis, TGF-β plays a crucial role regulating thyrocyte growth and differentiation, together with the thyroid-stimulating hormone [44]. Additionally, the TGF-β pathway plays important roles in other cellular functions, i.e. apoptosis and cell motility [15, 44, 45].

TGF-β is synthesized as an inactive precursor that can be activated by different proteases produced by thyrocytes, antagonizing the mitotic effect of other growth factors and hormones [44]. Briefly, the canonical signaling pathway starts with TGF-β binding to its specific receptor type II, which phosphorylates and activates type I receptor (TBRI). TBRI propagates the signal through the activation and phosphorylation of cytoplasmic proteins known as R-SMADs (SMAD2 and SMAD3). SMAD4 combines the R-SMADs and directs this complex to the nucleus, where it will modulate transcription of the target genes. In thyroid tumors, similarly to some other carcinomas, the sensitivity to TGF-β is lost during tumor development [45]. SMAD4 deficiency has been widely associated with TGF-β resistance of tumor cells, contributing to accelerate the malignant progression [44, 45]. This fact is not considered essential for tumor initiation, but it has been proven that in its absence, the development of a more aggressive phenotype occurs [31, 46].

Our hypothesis is that miR-146-5p contributes to regulate cell migration and invasion through the targeting of SMAD4 in normal and tumor thyroid cell lines. Reinforcing this hypothesis, D’Inzeo et al. [47] have shown that in TPC-1 and BCPAP cells, characterized by a significant reduction in the level of SMAD4 protein, the overexpression of SMAD4 partially reestablishes TGF-β responsiveness and significantly reduces migration, showing that SMAD4 is a critical regulator of this process. The overexpression of SMAD4 in BCPAP cells significantly decreased migration and invasion to a degree very similar to that observed with the antagomiR 146-5p, suggesting that SMAD4 is, indeed, a very important target of miR-146b-5p in those cells, regulating cell migration.

## Conclusion

Our findings improve the understanding of the functional role of miR-146b-5p in thyroid gland oncogenesis. The regulatory capacity of miR-146b-5p on tumor invasion, possibly through SMAD4 and the impairment of TGF-β signaling, shows its important role on PTC aggressiveness and invasiveness. This study also shows that the role of miR-146b-5p is independent of the cell’s capacity to degrade ECM.

## Methods

### Cell lines and cell culture

Non tumor rat follicular cells (PCCL3) and human papillary thyroid carcinoma cell lines TPC-1 (spontaneously harboring RET/PTC-1 mutation) and BCPAP (BRAF^V^^600E^ oncogene) were provided by Professor Edna T. Kimura. Culture conditions and supplements for each cell line were performed as previously described [48]. Briefly, PCCl3 were cultured in Coon's F12 medium supplemented with 5 % fetal bovine serum (FBS), penicillin (100 U / ml) and streptomycin (100 mg / ml), amphotericin (1 μg/ml) and thyroid stimulating hormone stimulant (1U / ml), bovine transferrin (5 g / ml), hydrocortisone (10 nM) and insulin (10 / ml). TPC-1 and BCPAP were cultured in DMEM medium supplemented with 5 % and 10 % FBS respectively, 100 U / ml penicillin, 1 μg/ml streptomycin and 100 μg/ml amphotericin at 37 °C and 5 % CO_2_ atmosphere.

### Plasmids and transfections

PCCl3 were transfected with a pcDNA3.1 plasmid containing the genomic region of miR-146b-5p (PC-CMV-146b) or the pcDNA3.1 empty vector (PC-CMV- ø as control), as previously described [31]. Inhibition of miR-146b-5p in PCCl3 and PTC cell lines (highly expressed) was obtained with the use of antagomiR-146b-5p (30nM -Anti-miR ^TM^ miRNA Inhibitor Product Anti-hsa-miR-146b-5p, AM10105, Applied Biosystems, Foster City, CA, USA), transfected with Lipofectamine 2000. The overexpression of miR-146b-5p of PTC cell lines was performed by mimics miR-146b-5p (50nM, MirVana miRNA mimic has-miR-146b-5p, MC10105) transfection.

As controls, all cell lines were used in regular culture conditions, incubated with transfection reagent alone (Mock) or transfected with a commercially available negative control (Negative) (anti-miR Negative control#1, AM17010; MirVana miRNA mimic negative Control#1, 4464058, Applied Biosystems).

BCPAP-pBabe SMAD4 and BCPAP-pBabe-puro were created by transfecting the plasmids pBabe-puro-Smad4-Flag and pBABE-puro plasmids, respectively, into BCPAP cells. pBabe-puro-Smad4-Flag was a gift from Sam Thiagalingam (Addgene plasmid # 37041) and pBABE-puro was a gift from Hartmut Land & Jay Morgenstern & Bob Weinberg (Addgene plasmid # 1764) [48, 49].

### Quantitative PCR and miR-146b-5p mature miR quantification

Total RNA from cell lines was isolated with Trizol, according to the manufacturer's protocol (Invitrogen). For miR expression analysis, the TaqMan microRNA Reverse Transcription kit and RT Primers provided with the miR-146b-5p Taqman miR Assay (PN4373178; Applied Biosystems) were used according to the manufacturer’s instructions to cDNA synthesis from total RNA (10 ng). Subsequently, miR-146b-5p expression was detected from the cDNA product using TaqMan Universal PCR Master Mix No AmpErase UNG (Applied Biosystems) and Taqman miR Assay according to the manufacturer’s instructions (Applied Biosystems, Weiterstadt, Germany) by qPCR. As housekeeping controls, small nucleolar RNA - snoRNA (PN4427975; Applied Biosystems) and RNU6B (PN4427975, Applied Biosystems) were used for rat and human miR normalization, respectively.

For SMAD4 expression analysis, 1 μg of total RNA was reverse transcribed using M-MLV Reverse Transcription (Invitrogen) according to the Manufacturer’s protocol, and PCR product was amplified from cDNA using 1X SYBR Green Universal Master Mix (Applied Biosystems) and specific primer. RPL19 was used as an endogenous control from mRNA normalization. Data were acquired using ABI 7300 Real-Time PCR System (Applied Biosystems) and analyzed using the Q-Gene Program.

### Migration and invasion assays

Migration and invasion assays were performed using transwell chambers with membrane pore size of 8.0 μm (Corning Inc). Membranes were uncoated for the migration assays and coated with 25 μg Matrigel® (BD Biosciences) for invasion assays. They were incubated with PBS (migration) or Matrigel® during 1 h at 37 °C, 5 % CO_2_ atmosphere. About 5 × 10^4^ cells were suspended in culture medium containing 0,5 %-1 % FBS and plated in the upper chamber, whereas the lower chamber contained culture medium with 5 %-10 % FBS. After 8 h (TPC-1 and BCPAP cell lines) or 24 h (PCCl3 cell line) at 37 °C, 5 % CO_2_, non-migrating cells on the top chamber were removed using a cotton swab, and cells that migrated through the membrane were fixed (4 % paraformaldehyde - PFA in PBS) and stained with 0.5 % Crystal Violet. Cells were photographed using a Nikon Eclipse E600 microscope equipped with optical camera CF160 epi-fluorescence and counted (10 representative fields).

### Growth curve

Non tumor rat follicular thyroid cells (10 × 10^4^) were seeded in 35 mm Petri dishes and cultured for 24, 48 and 72 h and after antagomiR transfection, PTC (TPC-I and BCPAP) cell lines (1 x10^4^) were seeded after 40 h (0 h) and cultured for 8 and 24 h. At each time point the cells were trypsinized, collected, stained with Trypan Blue and the viable cells were counted using a Neubauer chamber. Each assay was performed in triplicate and repeated 3 times for each sample.

### Analysis of the F-actin cytoskeleton and time-lapse

Cells were seeded on glass coverslips (18 mm) with or without Matrigel® coating (10 μg/ml), positioned in 12-well plates and cultured for 8 h (PTC cell lines) or 24 h (PCCl3). Cells were then fixed and permeabilized with 4 % PFA containing 0.5 % Triton X-100 and 5 % sucrose, in PHEM buffer (25 mM Hepes, 10 mM EGTA, 2 mM MgCl_2_, 60 mM Pipes, pH 6,9) for 5 min, and post-fixed with 4 % PFA containing 5 % sucrose in PHEM buffer for 30 min at room temperature. F-actin was stained with phalloidin-Alexa 488 or rhodamine (Invitrogen) (1:500) for 1 h. After three washes (PHEM/100 mM glycine), the coverslips were mounted with Vectashield containing DAPI, for nuclei staining. Images were obtained using a Zeiss LSM780 Confocal Microscope with a Multiphoton laser (Spectraphysics), at the Central Facility CEFAP-USP. Objectives C-Apochromat 63x/1.2 W Corr M27 (D = 0.14–0.19 mm) (WD = 0.28 mm at D = 0.17 mm) and 40x/1.2 W (D = 0.14–0.19 mm) (WD = 0.28 mm at D = 0.17 mm) were employed. Live cells plated on uncoated or Matrigel®-coated glass-bottomed dishes were observed under phase contrast during 16 h using the same equipment, at 37 °C and 5 % CO_2_.

### Fluorescent substrate degradation assay

After 40 h of antagomiR-146b-5p transfection, PTC cell lines were plated on coverslips coated with gelatin-FITC (Invitrogen) and cultured for 24 h. Cells were then fixed and F-actin was stained as previously described. F-actin and gelatin degradation areas were analyzed and photographed using a Zeiss LSM780 Confocal Microscope with a Multiphoton laser (Spectraphysics), at the Central Facility CEFAP-USP. Substrate degraded areas were measured using the Image J public software (NIH). The same assays were performed after overexpression of miR-146b-5p in both tumor cell lines for 8 h.

### Protein expression analyses

BCPAP-pBabe SMAD4 and BCPAP-pBabe-puro were seeded (1 × 10^6^ cells), tripsinized and lysed in presence of cocktail proteases inhibitors. Total protein (50 μg) were separated by 10 % polyacrilamide gel electrophoresis (PAGE) and transferred onto nitrocellulose membranes (Cat # 162–0115, Bio Rad). Membranes were incubated with monoclonal anti-SMAD4 antibody (sc-7966) from Santa Cruz Biotecnology Inc, and visualized using an Enhanced ChemoLuminescence kit (Clarity TM Western ECL Substrate, Cat # 170–5061, Bio Rad), according to the manufacturer’s instructions. The polyclonal anti-alpha tubulin antibody (ab4074, ABCAM) was used to normalize the protein expression.

### Statistical analysis

The GraphPad Prism (version 5.0) program was used for statistical analysis. Student’s *t*-test or two-way ANOVA followed by Tukey’s post-test were used, according to the comparison. Differences were considered statistically significant at *P* < 0.05.

## Additional files

Additional file 1: Figure S1. Overexpression of miR-146b-5p increases migration and invasion of the PTC cell lines, TPC-1 and BCPAP. Cells were transfected with an oligonucleotide mimics miR-146b-5p (miR-146b) (50nM), as described in the Methods section. Three control groups were used: (1) cells cultured in regular culture medium (identified as TPC-1, BCPAP), (2) cells incubated with the transfection agent only (Mock) and (3) cells transfected with a negative miR-control (Neg). Forty-eight hours after transfection miR-146b-5p expression (A, D) was evaluated. Transwell migration (without basement membrane) and invasion (with basement membrane) assays were performed during 8 h. Quantitative data are shown for migration (B, E) and invasion assays (C, F). All experiments were performed three times. TPC-1/BCPAP: cell, Mock: cell + transfection agent, Neg: cell + mimics-miR negative control, miR-146b: cell + mimics miR-146b-5p. Statistically significant differences: * *P* < 0,01 (TPC-1/BCPAP versus miR-146b); ** *P* < 0,01 (Mock versus miR-146b), *** *P* > 0,01 (Neg versus miR-146b). (TIF 521 kb) Additional file 2: Figure S2. Overexpression of miR-146b-5p slightly increases gelatin degradation by TPC-1 cells. Forty hours after transfection, cells were seeded upon glass coverslips (18 mm) coated with fluorescent gelatin and cultured for 8 h. After this period, cells were fixed, stained for F-actin and nucleus. Images (30) were obtained using fluorescence microscopy (60x objective) and cells with and without degradation areas were counted. Data are shown for TPC-1 (A) and BCPAP (B) cells. The degradation activity of control and treated groups (miR-146b-5p) were identified as dark areas on gelatin-FITC background. TPC-1 / BCPAP: cell, Mock: cell + transfection agent, Neg: cell + mimics miR negative control, miR-146b: cell + mimics-miR-146b. (TIF 279 kb) Additional file 3: Figure S3. Overexpression of miR-146b-5p in TPC-1 and BCPAP cells does not affect morphology and F-actin distribution. Forty hours after transfection, cells were seeded upon glass coverslips (18 mm) without and with Matrigel® coating (10 μg/ml) and cultured for 8 h. After this period, cells were fixed, stained for F-actin (green) and nucleus (blue) and analyzed by confocal microscopy. Representative images of TPC-1 and BCPAP cells are shown. Cells are polarized and show one or two predominant lamellipodia. TPC-1/BCPAP: cell, Mock: cell + transfection agent, Neg: cell + mimics-miR negative control, miR-146b: cell + mimics-miR-146b. Bars: 10 μm. (TIF 1884 kb) Additional file 4: Figure S4. Inhibition of miR-146b-5p decreases migration and invasion of the non tumor rat thyroid follicular cell line (PCCl3). Cells were transfected with an oligonucleotide antagomiR-146b-5p (Anti-146b) (0.5 and 1nM), as described in the Methods section. Three control groups were used: (1) cells cultured in regular culture medium (identified as PCCl3), (2) cells incubated with the transfection agent only (Mock) and (3) cells transfected with a negative miR-control (Neg). Sixty-four hours after transfection miR-146b-5p expression (A) was evaluated. Transwell migration (without basement membrane) and invasion (with basement membrane) assays were performed during 24 h, forty hours after transfection. Quantitative data are shown for migration (B) and invasion assays (C). PCCl3: cell, Mock: cell + transfection agent, Neg: cell + anti-miR negative control, Anti-146b: cell + anti-miR-146b-5p. Statistically significant differences: * *P* < 0,05 (PCCl3 versus Anti-146b-1nM); ** *P* <0,05 (Mock versus Anti-146b-1nM), *** *P* < 0,05 (Neg versus Anti-146b-1nM). (TIF 269 kb)
