# Supplementary figures and images for: MiRNA-146b-5p upregulates migration and invasion of different Papillary Thyroid Carcinoma cells
Source: BMC Cancer. 2016 Feb 16;16:108. doi: 10.1186/s12885-016-2146-z (PMC4754828; doi:10.1186/s12885-016-2146-z)

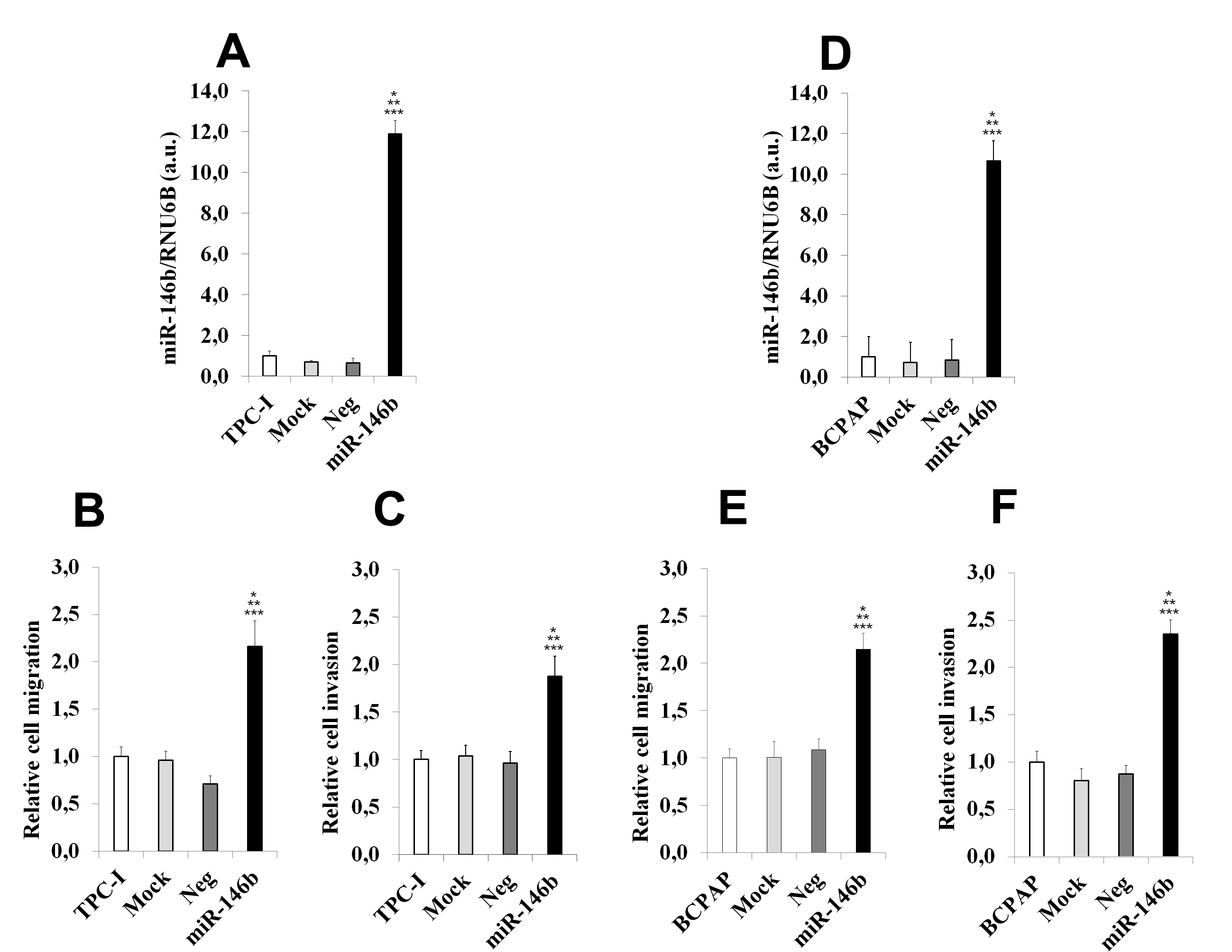

Supplement: Additional file 1: Figure S1. — Overexpression of miR-146b-5p increases migration and invasion of the PTC cell lines, TPC-1 and BCPAP. Cells were transfected with an oligonucleotide mimics miR-146b-5p (miR-146b) (50nM), as described in the Methods section. Three control groups were used: (1) cells cultured in regular culture medium (identified as TPC-1, BCPAP), (2) cells incubated with the transfection agent only (Mock) and (3) cells transfected with a negative miR-control (Neg). Forty-eight hours after transfection miR-146b-5p expression (A, D) was evaluated. Transwell migration (without basement membrane) and invasion (with basement membrane) assays were performed during 8 h. Quantitative data are shown for migration (B, E) and invasion assays (C, F). All experiments were performed three times. TPC-1/BCPAP: cell, Mock: cell + transfection agent, Neg: cell + mimics-miR negative control, miR-146b: cell + mimics miR-146b-5p. Statistically significant differences: * P < 0,01 (TPC-1/BCPAP versus miR-146b); ** P < 0,01 (Mock versus miR-146b), *** P > 0,01 (Neg versus miR-146b). (TIF 521 kb) [file 12885_2016_2146_MOESM1_ESM.tif]

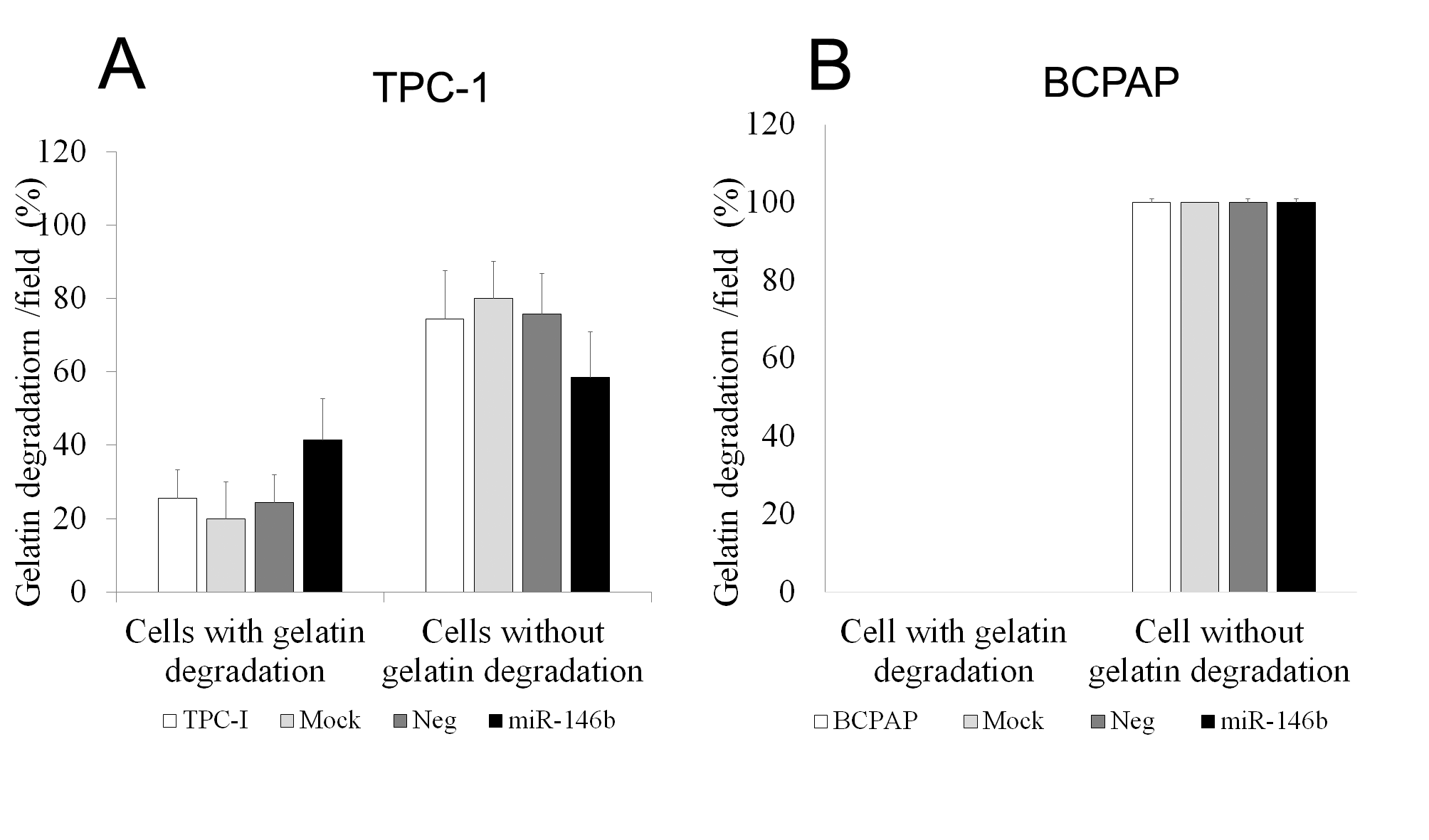

Supplement: Additional file 2: Figure S2. — Overexpression of miR-146b-5p slightly increases gelatin degradation by TPC-1 cells. Forty hours after transfection, cells were seeded upon glass coverslips (18 mm) coated with fluorescent gelatin and cultured for 8 h. After this period, cells were fixed, stained for F-actin and nucleus. Images (30) were obtained using fluorescence microscopy (60x objective) and cells with and without degradation areas were counted. Data are shown for TPC-1 (A) and BCPAP (B) cells. The degradation activity of control and treated groups (miR-146b-5p) were identified as dark areas on gelatin-FITC background. TPC-1 / BCPAP: cell, Mock: cell + transfection agent, Neg: cell + mimics miR negative control, miR-146b: cell + mimics-miR-146b. (TIF 279 kb) [file 12885_2016_2146_MOESM2_ESM.tif]

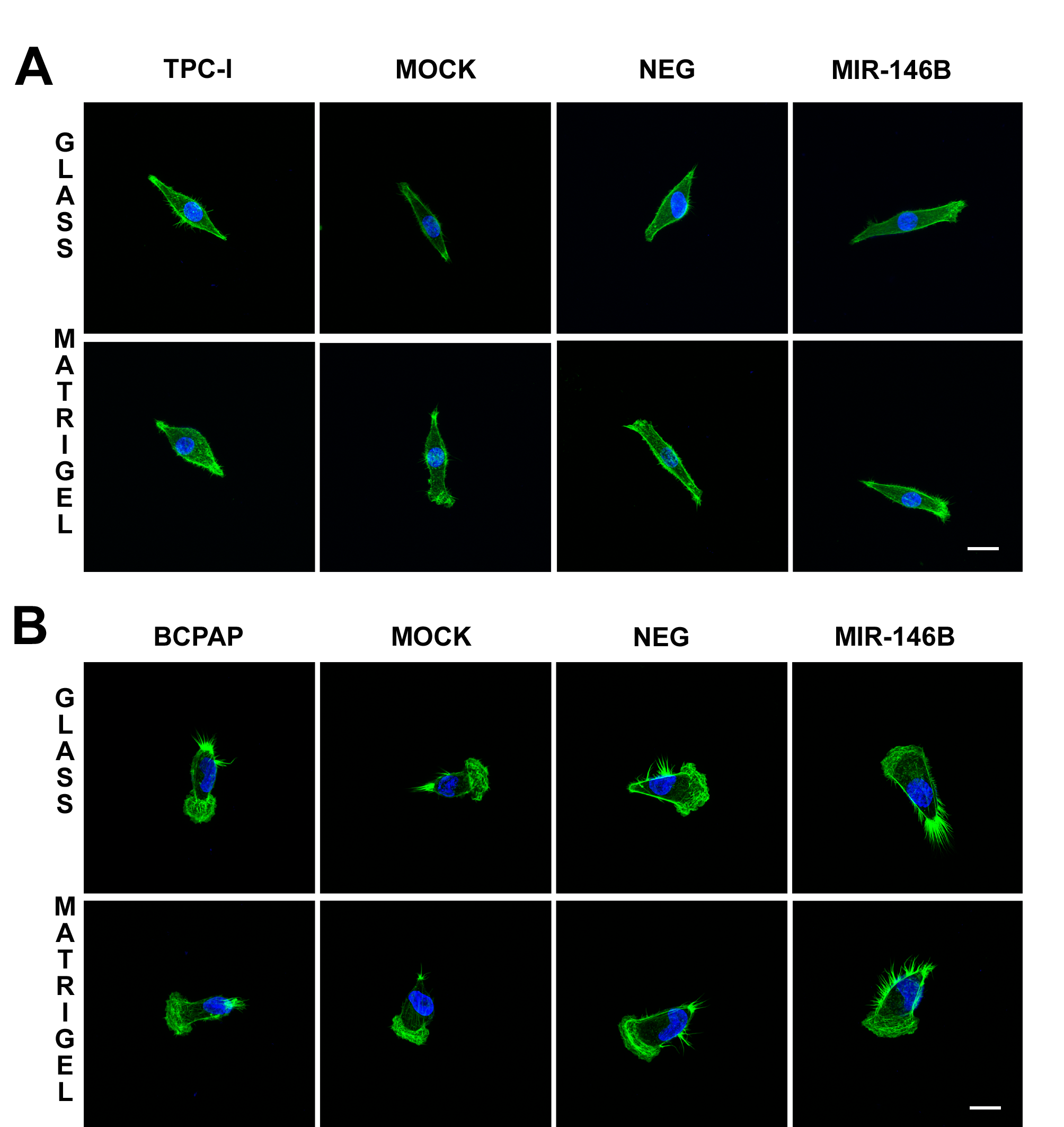

Supplement: Additional file 3: Figure S3. — Overexpression of miR-146b-5p in TPC-1 and BCPAP cells does not affect morphology and F-actin distribution. Forty hours after transfection, cells were seeded upon glass coverslips (18 mm) without and with Matrigel® coating (10 μg/ml) and cultured for 8 h. After this period, cells were fixed, stained for F-actin (green) and nucleus (blue) and analyzed by confocal microscopy. Representative images of TPC-1 and BCPAP cells are shown. Cells are polarized and show one or two predominant lamellipodia. TPC-1/BCPAP: cell, Mock: cell + transfection agent, Neg: cell + mimics-miR negative control, miR-146b: cell + mimics-miR-146b. Bars: 10 μm. (TIF 1884 kb) [file 12885_2016_2146_MOESM3_ESM.tif]

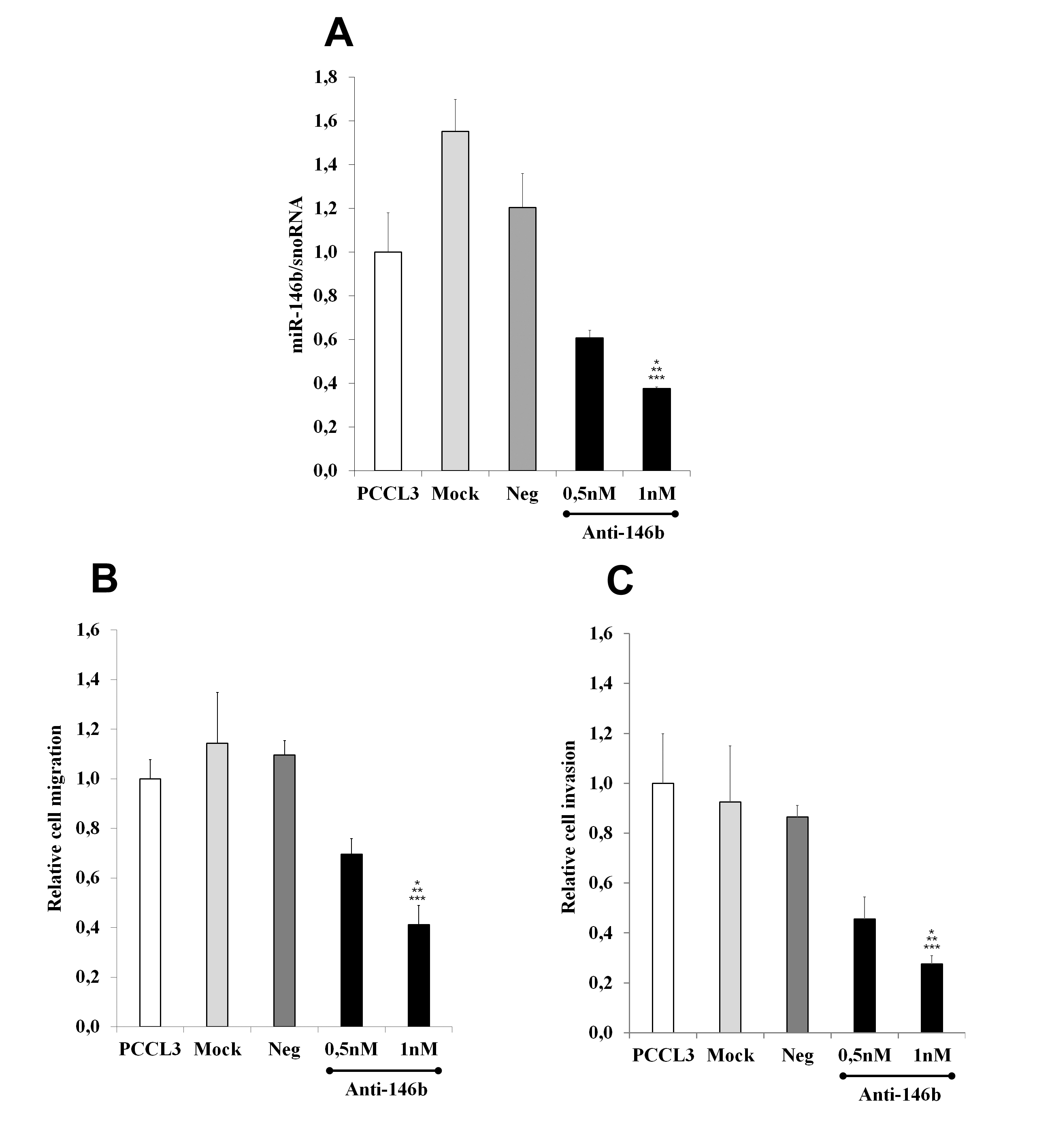

Supplement: Additional file 4: Figure S4. — Inhibition of miR-146b-5p decreases migration and invasion of the non tumor rat thyroid follicular cell line (PCCl3). Cells were transfected with an oligonucleotide antagomiR-146b-5p (Anti-146b) (0.5 and 1nM), as described in the Methods section. Three control groups were used: (1) cells cultured in regular culture medium (identified as PCCl3), (2) cells incubated with the transfection agent only (Mock) and (3) cells transfected with a negative miR-control (Neg). Sixty-four hours after transfection miR-146b-5p expression (A) was evaluated. Transwell migration (without basement membrane) and invasion (with basement membrane) assays were performed during 24 h, forty hours after transfection. Quantitative data are shown for migration (B) and invasion assays (C). PCCl3: cell, Mock: cell + transfection agent, Neg: cell + anti-miR negative control, Anti-146b: cell + anti-miR-146b-5p. Statistically significant differences: * P < 0,05 (PCCl3 versus Anti-146b-1nM); ** P <0,05 (Mock versus Anti-146b-1nM), *** P < 0,05 (Neg versus Anti-146b-1nM). (TIF 269 kb) [file 12885_2016_2146_MOESM4_ESM.tif]
